# Supplementary material for: Observation of dissipative chlorophyll-to-carotenoid energy transfer in light-harvesting complex II in membrane nanodiscs
Source: Nat Commun. 2020 Mar 10;11:1295. doi: 10.1038/s41467-020-15074-6 (PMC7064482; doi:10.1038/s41467-020-15074-6)
Supplement: Supplementary file 1 — Supplementary Information [file 41467_2020_15074_MOESM1_ESM.pdf]

Observation of dissipative chlorophyll-to-carotenoid energy transfer in light-harvesting complex II in membrane nanodiscs

Minjung Son[1], Alberta Pinnola[2,3], Samuel C. Gordon[1,5], Roberto Bassi[2,4], and Gabriela S. Schlau-Cohen[1,]

[1]*Department of Chemistry, Massachusetts Institute of Technology, 77 Massachusetts Avenue, Cambridge, Massachusetts 02139, USA*

[2]*Department of Biotechnology, University of Verona, Strada le Grazie 15, 37134, Verona, Italy*

[3]*Department of Biology and Biotechnology "L. Spallanzani", University of Pavia, Via A. Ferrata 1, 27100, Pavia, Italy*

[4]*Consiglio Nazionale delle Ricerche (CNR), Istituto per la Protezione delle Piante (IPP), Via Madonna del Piano 10, 50019, Sesto Fiorentino, Firenze, Italy*

[5]*Present address: Agenus Inc., 3 Forbes Road, Lexington, MA 02421, USA*

Contents

Supplementary Note 1. Detailed description of sample preparation 3

Supplementary Note 2. Additional experimental methods 10

Supplementary Note 3. Supplementary characterization data of LHCII membrane discs 15

Supplementary Note 4. Linear absorption data 17

Supplementary Note 5. Supplementary CD data 19

Supplementary Note 6. Fluorescence data 20

Supplementary Note 7. Supplementary 2DES data 22

Supplementary Note 8. Kinetic models 32

Supplementary References 35

Supplementary Note 1. Detailed description of sample preparation

1.1 Isolation and purification of LHCII

Thylakoid isolation. Stacked thylakoids were purified from leaves of spinach plants following a previously reported protocol[1] with minor modifications. Tissues were harvested and freshly homogenized in cold extraction buffer (0.5% milk powder, 0.4 M NaCl, 0.02 M Tricine/KOH, pH 7.8, 0.002 M $MgCl_2$ and 0.005 M ε-aminocaproic acid, 0.001 M phenylmethylsulfonyl fluoride and 0.001 M benzamidine as protease inhibitors). After filtration, samples were precipitated by centrifugation at 4,000 $\times$ g for 10 min at 4°C and then resuspended in hypotonic buffer (0.015 M NaCl, 0.005 M $MgCl_2$, 0.01 M HEPES/KOH, pH 7.5, and protease inhibitor). After centrifugation for 10 min at 10,000 $\times$ g at 4°C, thylakoids were resuspended in a buffer containing 0.4 M sorbitol, 0.015 M NaCl, 0.005 M $MgCl_2$, and 0.01 M HEPES/KOH, pH 7.5.

Pigment-binding complex purification. For separation of pigment-binding complex (LHCII), 3 mg Chl of thylakoid membranes were washed with 5 mM EDTA and resuspended at a final concentration of 1 mg mL^{-1} Chl in 0.01 M HEPES, pH 7.5. Samples were then solubilized at a final concentration of 0.5 mg mL^{-1} Chl, adding 1.6% *n*-dodecyl α-D-maltopyranoside (α-DM) and 0.01 M HEPES, pH 7.5 and vortexing for 1 min. After 10 min of incubation on ice, thylakoid membranes were centrifuged at 15,000 $\times$ g for 10 min to eliminate unsolubilized material. Fractionation occurred upon ultracentrifugation on a 0.1$-$1 M sucrose gradient containing 0.03% α-DM and 0.01 M HEPES, pH 7.5 (40 h at 103,000 $\times$ g at 4°C). The purified LHCII sample was stored at -80°C until further use, and thawed immediately before each laser measurement.

Pigment composition analysis. For pigment composition analysis, pigments were extracted with 80% acetone and centrifuged two times at 20,000 $\times$ g for 15 min at 4°C. The supernatant was analyzed by HPLC as described in Gilmore *et al.*[2] (Supplementary Figure 1). The pigment composition of purified LHCII determined is shown in Supplementary Table 1.

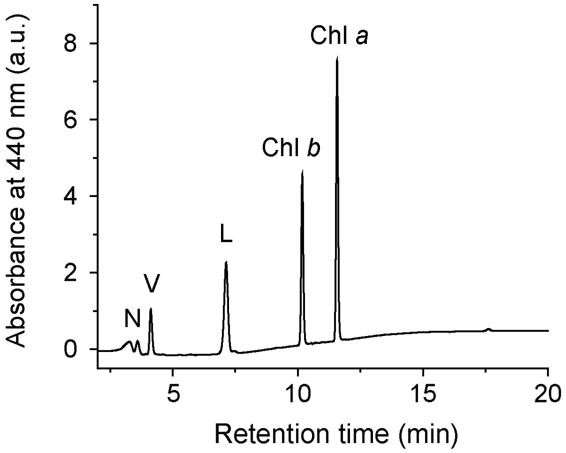

Supplementary Figure 1: HPLC chromatogram of the purified LHCII sample. HPLC profile of the purified LHCII sample measured at 440 nm. The determined pigment composition is given below in Supplementary Table 1. N: neoxanthin, V: violaxanthin, L: lutein.

Supplementary Table 1: Pigment composition of LHCII determined by HPLC.

Pigment	
Chl *a*/Chl *b*	1.26 ± 0.02
Chl *a* per mol	7.8 ± 0.09
Chl *b* per mol	6.2 ± 0.04
Neoxanthin	0.7 ± 0.05
Violaxanthin	0.6 ± 0.01
Lutein	2.0 ± 0.04
Zeaxanthin	0
Chl/Car	4.3 ± 0.12
Chl total	14
Car total	3.3 ± 0.09

1.2 Preparation and characterization of LHCII nanodiscs

1.2.1 Purification of membrane scaffold proteins

Purification of MSP1E3D1. The membrane scaffold protein MSP1E3D1 was overexpressed from the plasmid pMSP1E3D1 containing a 6× histidine tag and kanamycin antibiotic resistance (Addgene), and purified with nickel affinity chromatography according to previously reported protocols.[3,4] The mass and purity of the final product were verified by reverse-phase liquid chromatography mass spectrometry (LC-MS) and denaturing sodium dodecyl sulfate polyacrylamide gel electrophoresis (SDS-PAGE). The purified

protein was concentrated to ~600 μM and aliquoted. Protein concentration was determined using the absorbance of the protein at 280 nm and the known extinction coefficient of MSP1E3D1 at this wavelength.[4] The aliquots were flash frozen in liquid nitrogen and stored at -80°C until further use.

Purification of ApoE422K. ApoE422K membrane scaffold protein, the N-terminal 22 kDa fragment of human apolipoprotein E4, was overexpressed and purified following a previously reported protocol with minor modifications.[5] Briefly, thioredoxin-ApoE422K-full length fusion protein was overexpressed from the plasmid pD451-SR containing containing a 6$\times$ histidine tag and kanamycin antibiotic resistance (DNA 2.0), and purified with nickel affinity chromatography similarly to the purification of MSP1E3D1. After nickel affinity purification, thrombin (Sigma Aldrich) was added to the eluate for cleavage of thioredoxin, and the mixture was incubated overnight at room temperature. SDS-PAGE was used to monitor the yield of the cleavage reaction. Following complete cleavage, the product was purified once more with nickel affinity chromatography. The purified protein was concentrated to ~200 μM and aliquoted. Protein concentration was determined using the absorbance of the protein at 280 nm and the extinction coefficient of ApoE422K at this wavelength (30,940 $M^{-1}cm^{-1}$). The aliquots were flash frozen in liquid nitrogen and stored at -80°C until further use.

1.2.2 Preparation of lipid stocks

Preparation of lipids was performed similarly to the method reported in earlier work.[6, 7] Soy asolectin lipid mixture was purchased from Sigma Aldrich and used without further purification. A 25 mg mL^{-1} stock solution was prepared by solubilizing the lipid mixture in 0.05 M HEPES, 0.1 M NaCl, 0.04 M sodium cholate buffer (pH 7.5). The concentration of lipid was determined by phosphorus assay.[8] For nanodisc assembly with native thylakoid lipid mixture, the four lipid components monogalactosyldiacylglycerol (MGDG), digalactosyldiacylglycerol (DGDG), sulfoquinovosyldiacylglycerol (SQDG), and phosphatidylglycerol (PG) were purchased from Avanti Polar Lipids, and each solubilized to a 5 mg mL^{-1} concentration in 0.05 M HEPES, 0.1 M NaCl, 0.04 M sodium cholate buffer (pH 7.5). The prepared stock lipid solutions were aliquoted and degassed of oxygen by bubbling in N_2 gas. The aliquots were flash frozen and stored at -80°C until further use.

1.2.3 Preparation of LHCII nanodiscs

Assembly of 13 nm diameter LHCII nanodiscs with soy asolectin lipid. Nanodiscs loaded with LHCII were produced following a previous reported protocol with minor modifications.[6, 7] Purified MSP1E3D1, soy asolectin, and detergent-solubilized LHCII were mixed together at a molar ratio of 1 : 55 : 0.125. Excess MSP1E3D1 and lipid were used to minimize contamination of the sample with LHCII not embedded in the disc or multiply-embedded LHCII. The MSP1E3D1:lipid molar ratio was systematically varied to optimize

the yield, and an optimal molar ratio of 1 : 55 : 0.125 was determined. The mixture was incubated at 4°C shaking for 1 h. To remove the detergent, Bio-Beads SM-2 adsorbents (Bio-Rad) were added to the reaction and incubated at 4°C shaking for 1 h. Bio-Beads were removed by centrifugation at 4,000 × g for 20 min. The product was purified by the 6× histidine tag of MSP1E3D1 on a nickel affinity column. Following equilibration of the sample with the beads for 2 h at 4°C on a nutating mixer, column flow-through was collected and the column was washed with 0.01 M HEPES, 0.02 M NaCl, 0.02 M imidazole, pH 7.5. The sample was eluted with 0.01 M HEPES, 0.02 M NaCl, 0.4 M imidazole, pH 7.5 in fractions. Fractions containing loaded nanodiscs were determined by SDS-PAGE and linear absorption. No LHCII was detected in the flow-through or washes, confirming 100% incorporation of LHCII into discs. Imidazole in the purified discs was removed by buffer exchange into 0.01 M HEPES, 0.02 M NaCl, 0.5 mM EDTA, pH 7.5. The sample was further buffer exchanged into 0.05 M HEPES, 0.15 M NaCl, pH 7.5. The loaded nanodiscs were further purified by fast protein liquid chromatography (FPLC) with a BioLogic DuoFlow (Bio-Rad) on a Superdex 200 Increase 10/300 GL (GE Healthcare Life Sciences) at a flow rate of 0.75 mL min^{-1} in 0.05 M HEPES, 0.15 M NaCl, pH 7.5. Fractions of the main peak were collected and analyzed by linear absorption, SDS-PAGE, and transmission electron microscopy (TEM) to identify the peak containing LHCII discs. TEM samples were prepared by the negative staining method[9] with 2% uranyl acetate on negatively glow-discharged 400-mesh Cu-carbon coated films (Electron Microscopy Sciences). Samples were imaged on a FEI Tecnai (G2 Spirit TWIN) electron microscope operated at 120 kV.

Assembly of 13 nm diameter LHCII nanodiscs with native thylakoid lipids. To examine any dependence of the excited-state dynamics of LHCII on the lipids used, nanodiscs with lipid mixture consisting of native thylakoid lipids were also prepared. To mimic the lipid composition of the native plant thylakoid membrane (∼50% MGDG, ∼30% DGDG, ∼ 5 – 12% SQDG, and ∼ 5 – 12% PG)[10], lipid mixture containing 50% MGDG, 27% DGDG, 12% SQDG and 11% PG was prepared by mixing the stock aliquots prepared as described in Supplementary Note 1.2.2. Lipid mixture without the non-bilayer-forming lipid MGDG was also prepared (62% DGDG, 17% SQDG, 21% PG). Each lipid mixture was added to the disc reaction at the same molar ratio as in soy asolectin discs (1 : 55 : 0.125), and discs were prepared and characterized following the protocol described above for soy asolectin.

Assembly of 25 nm diameter LHCII nanodiscs with soy asolectin lipid. Larger-sized LHCII nanodiscs were prepared similarly to the 13 nm diameter nanodiscs. ApoE422K membrane scaffold protein was used instead of MSP1E3D1. Excess ApoE422K and lipid (soy asolectin) were used to minimize contamination of the sample with LHCII not embedded in the disc or multiply-embedded LHCII. Stoichiometry of the components was systematically varied to optimize the yield of the product, and an optimal molar ratio of ApoE422K : LHCII: lipid = 1 : 0.042 : 180 was determined. Incubation, removal of detergent, nickel

affinity purification, and buffer exchange were performed exactly as described for the 13 nm diameter nanodiscs. FPLC purification could not be performed because the sample stuck on the size exclusion column, resulting in $> 90\%$ loss of the product. Supplementary Figures 2−4 demonstrate successful incorporation of LHCII into nanodiscs. All 2D data presented in this manuscript were measured on LHCII nanodiscs with soy asolectin lipids and MSP1E3D1 membrane scaffold protein. The nanodisc samples were produced immediately before any spectroscopy measurement, and all spectroscopic measurements were completed within 24 hours of sample preparation.

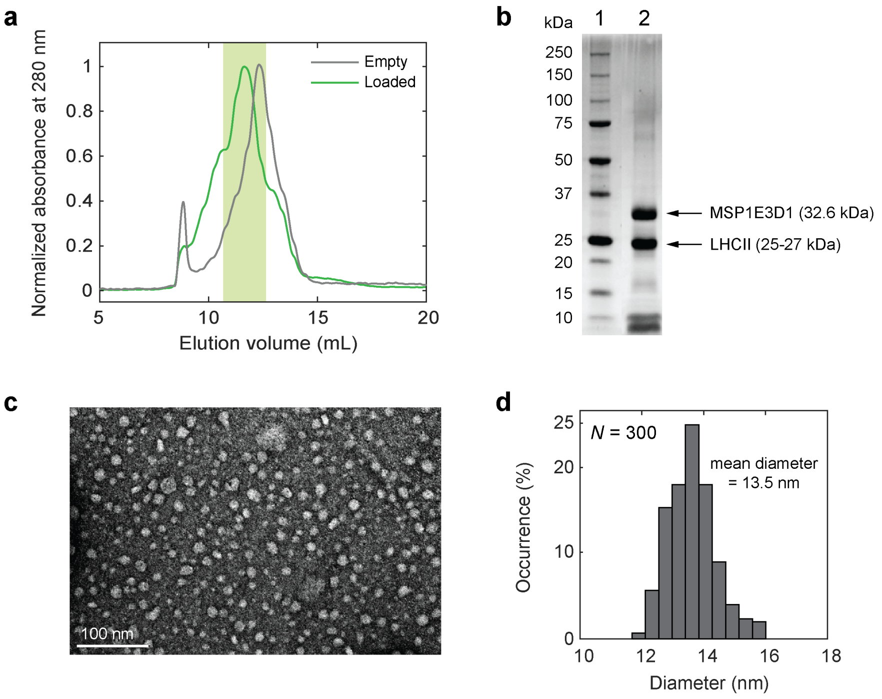

Supplementary Figure 2: Characterization of LHCII nanodiscs with soy asolectin lipid. a, FPLC chromatogram of empty (gray) and loaded (green) LHCII nanodisc with soy asolectin lipid. Green shaded area indicates the fraction of loaded discs that was stored for spectroscopic measurements. **b**, SDS-PAGE of loaded discs after FPLC purification. Lane 1: ladder, lane 2: LHCII discs. Both the MSP1E3D1 and LHCII bands are identified, which shows successful incorporation of LHCII into discs. **c-d**, TEM image (**c**) and size distribution for 10−18 nm objects (**d**) derived from TEM image analysis.

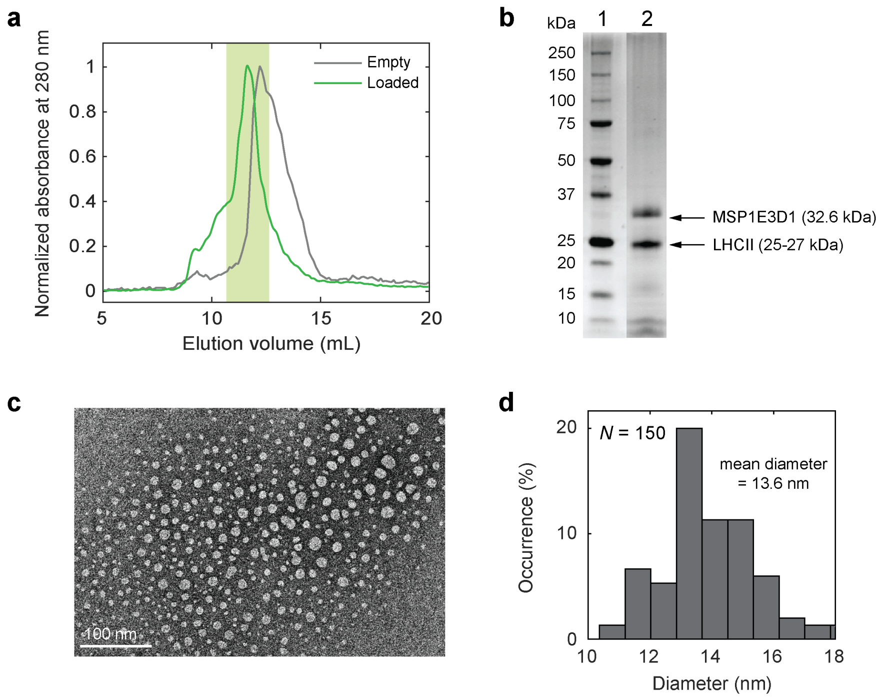

Supplementary Figure 3: Characterization of LHCII nanodiscs with thylakoid lipid mixture. a, FPLC chromatogram of empty (gray) and loaded (green) LHCII nanodisc with 50% MGDG, 27% DGDG, 12% SQDG and 11% PG. Green shaded area indicates the fraction of loaded discs that was stored for spectroscopic measurements. **b**, SDS-PAGE of loaded discs after FPLC purification. Lane 1: ladder, lane 2: LHCII discs. **c-d**, TEM image (**c**) and size distribution for 10−18 nm objects (**d**) derived from TEM image analysis.

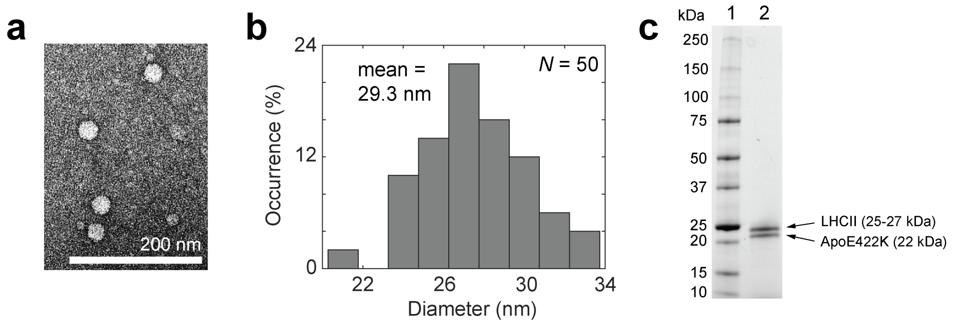

Supplementary Figure 4: Characterization of larger LHCII nanodiscs. a-b, Representative TEM image (**a**) and size distribution (**b**) of the discs derived from TEM image analysis. **c**, SDS-PAGE analysis of loaded discs after purification. Lane 1: ladder, lane 2: LHCII discs. Both the ApoE422K and LHCII bands are identified, which shows successful incorporation of LHCII into discs. Soy asolectin lipids are used.

1.3 Purity of the nanodiscs

As shown in Supplementary Figures 2b and 3b, the SDS-PAGE results show several low molecular weight bands below 25 kDa. These impurities originate from imperfect purification of the membrane scaffold protein MSP1E3D1 (Supplementary Figure 5), and are maintained during nanodisc preparation.[11] While the intensities of these bands vary in each preparation, they are typically $< 20\%$ of that of the main 32.6 kDa band. The linear absorption spectrum even in the presence of these impurities shows a single peak at 280 nm, originating from the absorption of aromatic amino acids, and no absorbance in the visible range, indicating that none of the photophysical data presented herein is affected by these impurities.

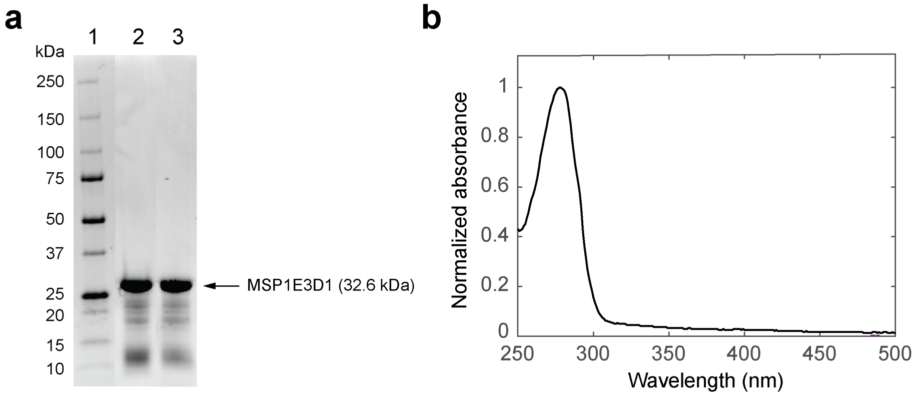

Supplementary Figure 5: Characterization of free MSP1E3D1 and empty nanodiscs. a, SDS-PAGE of free MSP1E3D1 protein (lane 2) and empty nanodiscs without any LHCII incorporated (lane 3). Lane 1: ladder. Samples for both lanes 2 and 3 were at saturating concentration ($\sim$100 μM protein, 5 times higher than the concentration used for Supplementary Figures 2b and 3b to clearly visualize the presence of impurity bands). **b**, Linear absorption of empty nanodiscs (lane 3 in **a**).

Supplementary Note 2. Additional experimental methods

2.1 Steady-state absorption and fluorescence

Linear absorption and steady-state fluorescence spectra were measured with a Cary 5000 spectrophotometer and a Cary Eclipse fluorimeter (Agilent), respectively, in 1 cm pathlength cuvettes. For fluorescence measurements, the optical density (OD) of the sample at the excitation wavelength was kept at $0.05 - 0.07$ to ensure absence of any reabsorption effect. Excitation wavelengths of 436 nm, 600 nm, and 640 nm were used, and no excitation-wavelength dependence was found in the fluorescence spectra. Relative fluorescence quantum yield of LHCII in nanodiscs compared to LHCII in detergent was calculated by comparing the integrated area of the fluorescence spectrum normalized by the OD at the excitation wavelength.

2.2 Circular dichroism (CD)

CD spectra were measured at 20°C on a Jasco J-1500 spectropolarimeter, in a 1 mm pathlength quartz cuvette. The OD of the sample was 0.08 per mm at 670 nm.

2.3 Time-correlated single photon counting (TCSPC)

The excitation source was generated by passing the output of a Ti:sapphire laser (Vitara-S, Coherent Inc., centered at 800 nm, 80-MHz repetition rate) through a nonlinear photonic crystal fiber (FemtoWhite 800, NKT Photonics). The generated broadband supercontinuum was then passed through a $630 - 655$ nm bandpass filter (ET645/30x, Chroma, Supplementary Figure 6) to produce the excitation laser spectrum. The excitation beam was focused on a 1-mm pathlength cuvette containing the sample and the emission from sample was first passed through an emission filter (665.2 nm longpass, BLP01-647R-25, Semrock), and then detected by a single-photon-detecting avalanche photodiode (PDM Series, Micro Photon Devices). The output of the detector was connected to a timing module (PicoHarp 300, PicoQuant, Inc.), which detects the arrival time of each photon. The instrument response function (IRF) was determined by measuring scattered excitation light, and has a width of 50 ps. Fluorescence decay curves were individually fitted with a mono-exponential or bi-exponential decay function with the IRF using iterative reconvolution. To prevent reabsorption effect, the OD of all samples at the excitation wavelength range was kept around 0.07 per mm. The excitation laser power used was 10 fJ per pulse for all samples.

2.4 Fluorescence correlation spectroscopy (FCS)

FCS measurements were performed on a home-built confocal microscope. The excitation source was generated by tuning a fiber laser (FemtoFiber pro, Toptica Photonics; 80 MHz repetition rate), and was centered at 610 nm with a 4 nm full-width at half maximum (FWHM) bandwidth. The fluorescence signal was isolated using a dichroic mirror (T635lpxr, Chroma) and a bandpass filter (ET700/75m, Chroma, Supplementary

Figure 6). Samples were diluted to 5 nM, and allowed to freely diffuse within a 25 mm diameter hybridization chamber sealed to a glass coverslip. Sample excitation and fluorescence collection were accomplished by the same oil-immersion objective (UPLSAPO100XO, Olympus; NA = 1.4). The excitation laser fluence on the sample plane was 504 nJ per cm^2. Fluorescence signal was detected using an avalanche photodiode (SPCM-AQRH-15, Excelitas). The diffusion time constant (τ_D) was determined by fitting the autocorrelation of the detected photons ($G(t)$, typically 2×10^6 photons were collected to construct each correlation curve) to equation (1),[12]

$$G(t) = C + \left(\frac{1}{N}\right)\left(\frac{1}{1+\frac{t}{\tau_D}}\right)\left(\frac{1}{\sqrt{1+\frac{t}{V^2\tau_D}}}\right)\left(1 - f_T + f_T e^{\frac{-t}{\tau_T}}\right) \tag{1}$$

where t is the correlation time, C is a constant, N is the number of molecules in the focal volume, τ_D is the diffusion time constant, V is a measure of the detection volume defined as Z_o/w_o, where Z_o and w_o are the effective half axial and radial dimensions of the focal volume, respectively, f_T is the fractional population of the triplet state, and τ_T is the triplet lifetime. The diffusion constant (D) and the hydrodynamic radius (r) of each sample were determined using equations (2) and (3) (the Stokes-Einstein relation):

$$\tau_D = \frac{{w_o}^2}{4D} \tag{2}$$

$$D = \frac{k_B T}{6\pi\eta r} \tag{3}$$

where k_B is the Boltzmann constant, T is the temperature (T = 20°C was used in this work), and η is the viscosity of the medium.

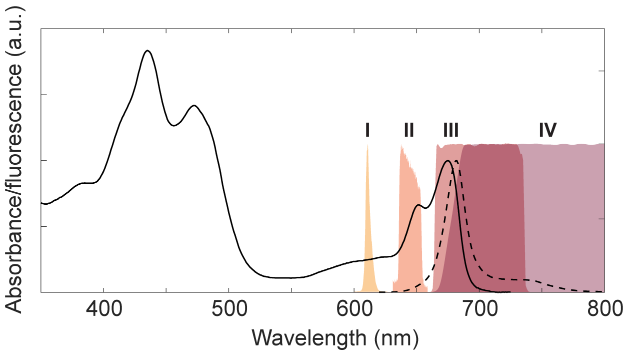

Supplementary Figure 6: Experimental condition for TCSPC and FCS measurements. Transmission spectra of the excitation (**I** for FCS, **II** for TCSPC) and emission (**III** for FCS, **IV** for TCSPC) filters overlaid with the absorption (black solid line) and fluorescence (black dashed line) spectra of LHCII.

2.5 Detailed description of two-dimensional electronic spectroscopy (2DES) apparatus

The ultrabroadband laser spectra were obtained by focusing the output of a Ti:sapphire regenerative amplifier (Libra, Coherent Inc., 5 kHz repetition rate, 1.1 mJ output, $<$ 40 fs pulse duration centered at 800 nm) into pressurized argon gas at 20 psi above atmospheric pressure. The generated supercontinuum was passed through a 805-nm cutoff dichroic mirror (Thorlabs) and glass bandpass filters (Thorlabs) to attenuate the near-infrared part. Glass filters with different cutoff wavelengths were chosen to tune the laser spectrum for optimal excitation of the Car S_2/Chl Q_x (spectrum 1) and Chl Q_x/Q_y regions (spectrum 2), respectively (Supplementary Figure 7a). Spectrum 1 was centered at 550 nm (18,182 cm^{-1}) with a FWHM of 113 nm (3,819 cm^{-1}), and spectrum 2 was centered at 614 nm (16,287 cm^{-1}) with a FWHM of 168 nm (4,807 cm^{-1}). The final spectra were compressed with group-velocity-dispersion (GVD)-compensated chirped mirror pairs (Ultrafast Innovations GmbH) to $6.2-6.9$ fs pulses as characterized by transient grating frequency-resolved optical gating (TG-FROG, Supplementary Figure 7b, c).[13]

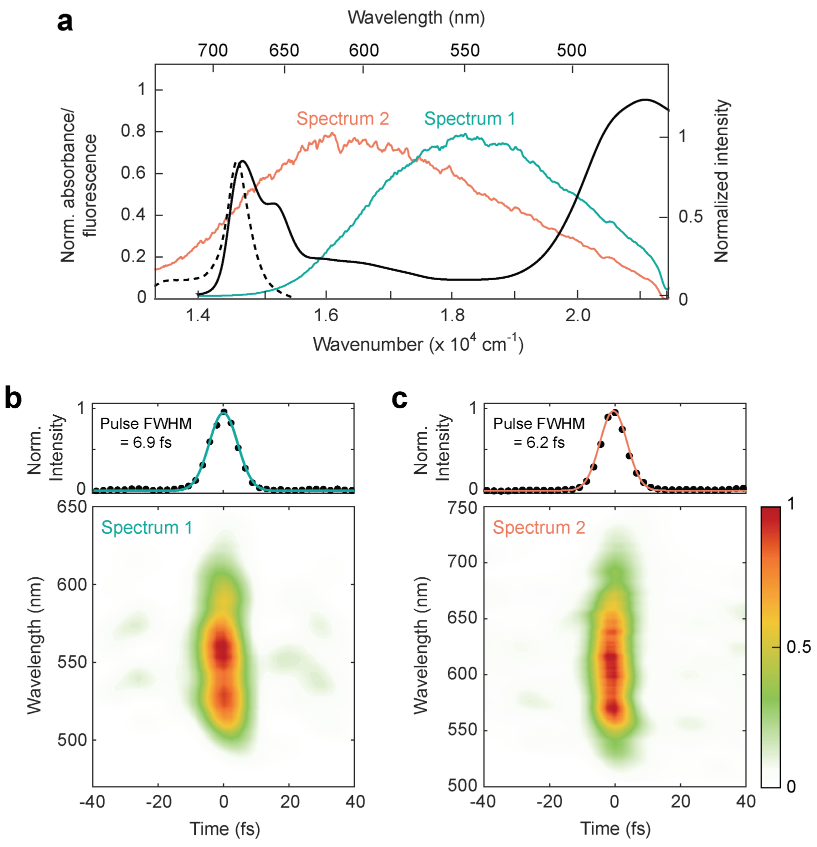

Supplementary Figure 7: Characterization of laser spectra used for 2DES measurements. a, Ultrabroadband laser spectra used in 2DES overlaid with the linear absorption (black solid) and fluorescence spectra (black dashed) of LHCII. **b-c**, TG-FROG trace of spectra 1 (**b**) and 2 (**c**) with the retrieved temporal profiles (filled circles) fitted with a Gaussian function (solid line).

The compressed pulse was split into four beams in the BOXCARS geometry. Temporal delays between pulses were achieved by two nanopositioners and a translational stage (Aerotech) to encode the coherence time (τ) and waiting time (T), respectively. The local oscillator was attenuated by three orders of magnitude and temporally delayed by $600-700$ fs from the three other pulses. The emitted third-order signal was heterodyne-detected by focusing onto a home-built spectrometer. For scatter removal, we employed shot-to-shot (5 kHz) data acquisition synchronized with dual chopping at 2.5- and 1.25 kHz frequencies, using a high-speed line scan CCD (e2v). At a given T, 1,280 lines for each chopper sequence were averaged and saved in the data matrix for each τ. For both datasets, τ was sampled in 0.4 fs steps in the range of $-200-200$ fs. T was incremented in steps of 10 fs for $T = 0-100$ fs, 33 fs for $T = 100-467$ fs, 67 fs for $T = 467$ fs $-$ 1 ps, and 1 ps for $T = 1-10$ ps. A pulse energy of 10 nJ was employed for all 2DES measurements. The detergent-solubilized and disc-embedded LHCII samples were measured consecutively under the same experimental condition for direct comparison of the dynamics. Each dataset was collected three times, on separate days, to ensure reproducibility of the data. Following each set of measurement, the integrity of the sample was confirmed by comparing the linear absorption spectra and fluorescence decay profiles before and after the experiment (Supplementary Figure 8). For phasing, auxiliary pump-probe spectra were collected shot-to-shot following each set of measurement, using only the 2.5-kHz chopper. The raw 2D spectra were phased using the pump-probe spectra according to the projection slice theorem.[14] Additional details of the 2DES apparatus and data acquisition can be found elsewhere.[15]

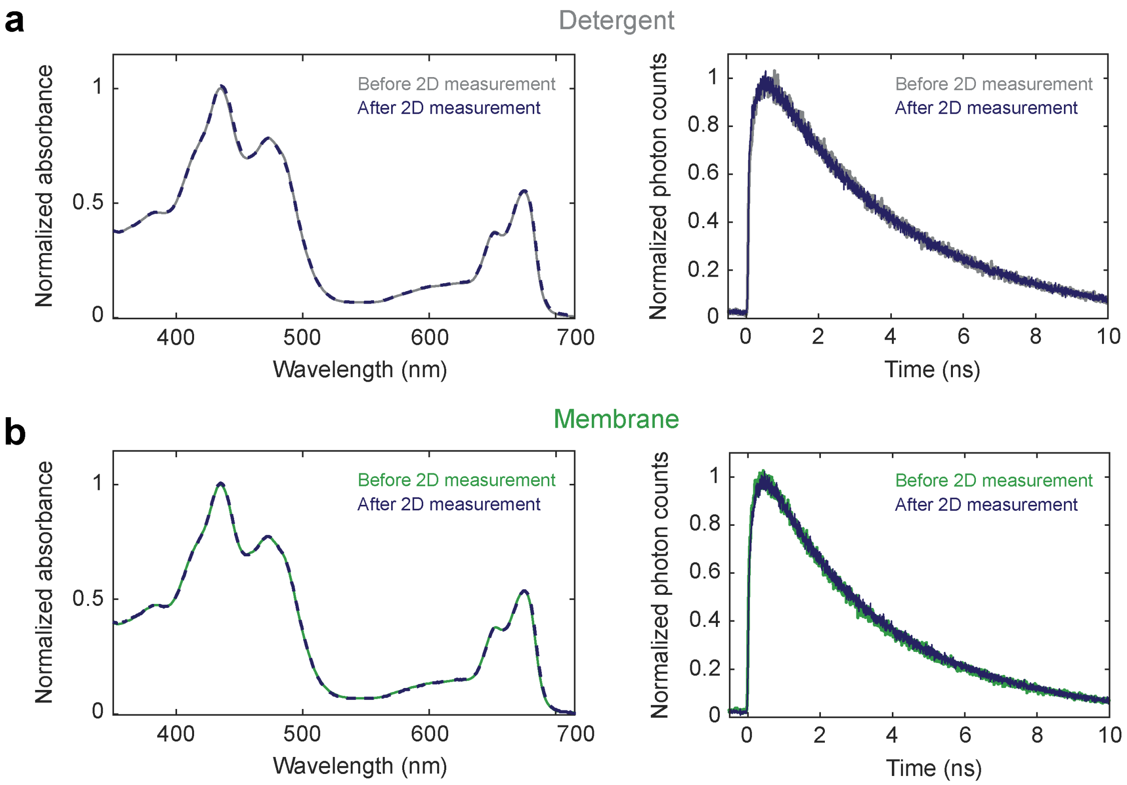

Supplementary Figure 8: Verification of sample integrity before and after 2DES measurements. Normalized linear absorption spectra (left) and fluorescence decay traces (right) for LHCII solubilized in detergent (**a**) and in the membrane (**b**), confirming the absence of sample degradation during 2DES measurements.

Supplementary Note 3. Supplementary characterization data of LHCII membrane discs

3.1 Absence of direct interactions between peripheral pigments of LHCII and the membrane scaffold protein

To evaluate the presence of direct interactions between the peripheral pigments in LHCII and the membrane scaffold protein due to the limited membrane surface area, we compared the photophysical properties of LHCII nanodiscs of two different sizes: $\sim$13 nm (formed with MSP1E3D1 membrane scaffold protein) and $\sim$25 nm diameter (formed with ApoE422K membrane scaffold protein, see Supplementary Note 1.2 and Supplementary Figure 4 for preparation and characterization of these discs).[16] As shown in Supplementary Figure 9 below, the absorption and fluorescence properties of LHCII nanodiscs are independent of the size of the nanodiscs, pointing to the interaction between LHCII and the lipid bilayer as the cause of the observed changes in the photophysics of LHCII upon membrane insertion.

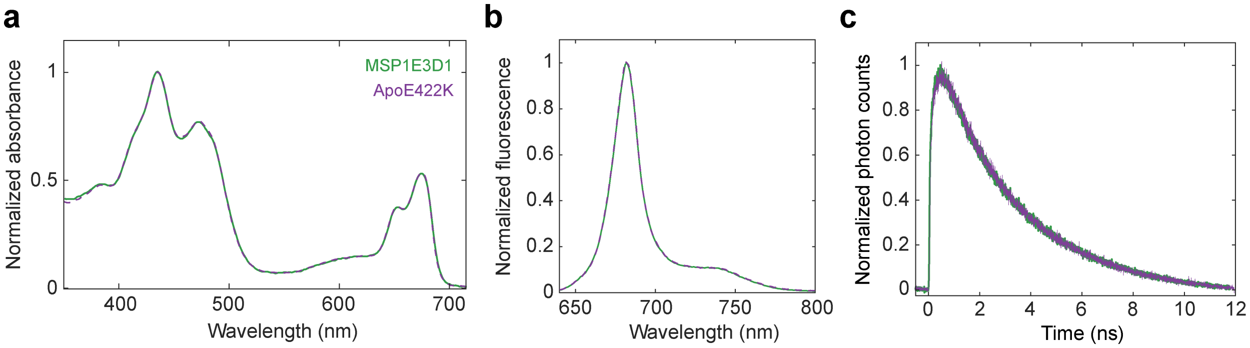

Supplementary Figure 9: Comparison of the photophysical properties of LHCII nanodiscs with MSP1E3D1 and ApoE422K membrane scaffold proteins. Normalized linear absorption spectra (**a**), fluorescence spectra (**b**), and fluorescence decay traces (**c**) are shown, with MSP1E3D1 nanodiscs as green and ApoE422K nanodiscs as purple (soy asolectin lipids were used in both cases).

3.2 Absence of contributions from LHCII self-aggregates

Absence of LHCII self-aggregates in the purified nanodisc sample, which are known to induce quenching of LHCII fluorescence,[17] was confirmed by FCS (see Supplementary Note 2.4 for details on experimental method and data analysis). As shown in Supplementary Figure 10, correlation curves in both environments are fitted with a single time component of 0.90±0.10 ms (detergent) and 1.52±0.20 ms (membrane), demonstrating the absence of LHCII aggregates in our nanodisc sample. The presence of LHCII aggregates would have resulted in poor fits with single components as well as much slower diffusion time constants (τ_D) due to the large size of the aggregates.

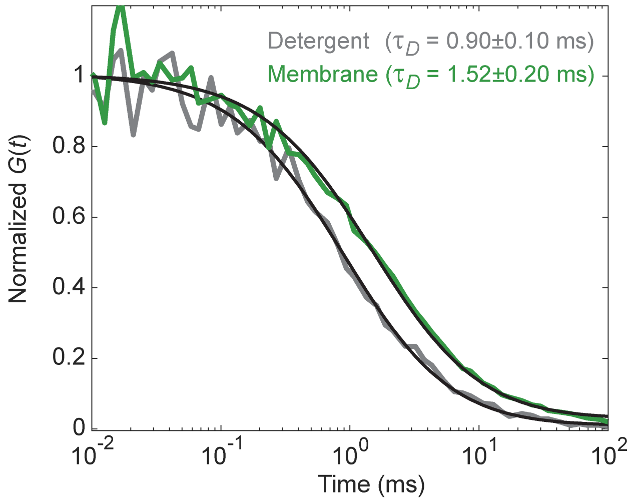

Supplementary Figure 10: Fluorescence correlation curves of detergent-solubilized and disc-embedded LHCII. Black curves are the fit curves. Both curves are normalized to the maximum G value at 10^{-2} ms, and the fitted diffusion constants (τ_D) with 95% confidence intervals are shown inside the figure.

Supplementary Note 4. Linear absorption data

4.1 Second-derivative analysis of absorption spectrum

Energy levels of the pigments were identified by taking the second derivative of the linear absorption spectrum of detergent-solubilized LHCII measured at 293 K and 77 K, and the data are reported in Son *et al.*[18] The identified transitions are summarized in Supplementary Table 2. Note that the S_2 states of Neo and Vio could not be assigned separately due to the significant overlap between the two states.[19]

Supplementary Table 2: Pigment energy levels from second-derivative analysis.

Transition	293 K wavelength (frequency)[a]	77 K wavelength (frequency)
Chl *a* Soret	433 (23,090)	431 (23,200)
Chl *b* Soret	473 (21,160)	473 (21,160)
Vio/Neo S_2	486 (20,580)	485 (20,600)
Lut1 S_2	n.d.[b]	493 (20,280)
Lut2 S_2	n.d.	511 (19,560)
Chl *b* Q_x	597 (16,750)	597 (16,750)
Chl *a* Q_x	621 (16,100)	621 (16,100)
Chl *b* Q_y	649 (15,400)	648 (15,430)
High-energy Chl *a* Q_y (Chl a_H)	n.d.	661 (15,130)
Low-energy Chl *a* Q_y (Chl a_L)	678 (14,750)	678 (14,750)

[a] Frequencies (in parentheses) are in cm^{-1}.

[b] Not determined.

4.2 Comparison of linear absorption in detergent and in the membrane environment

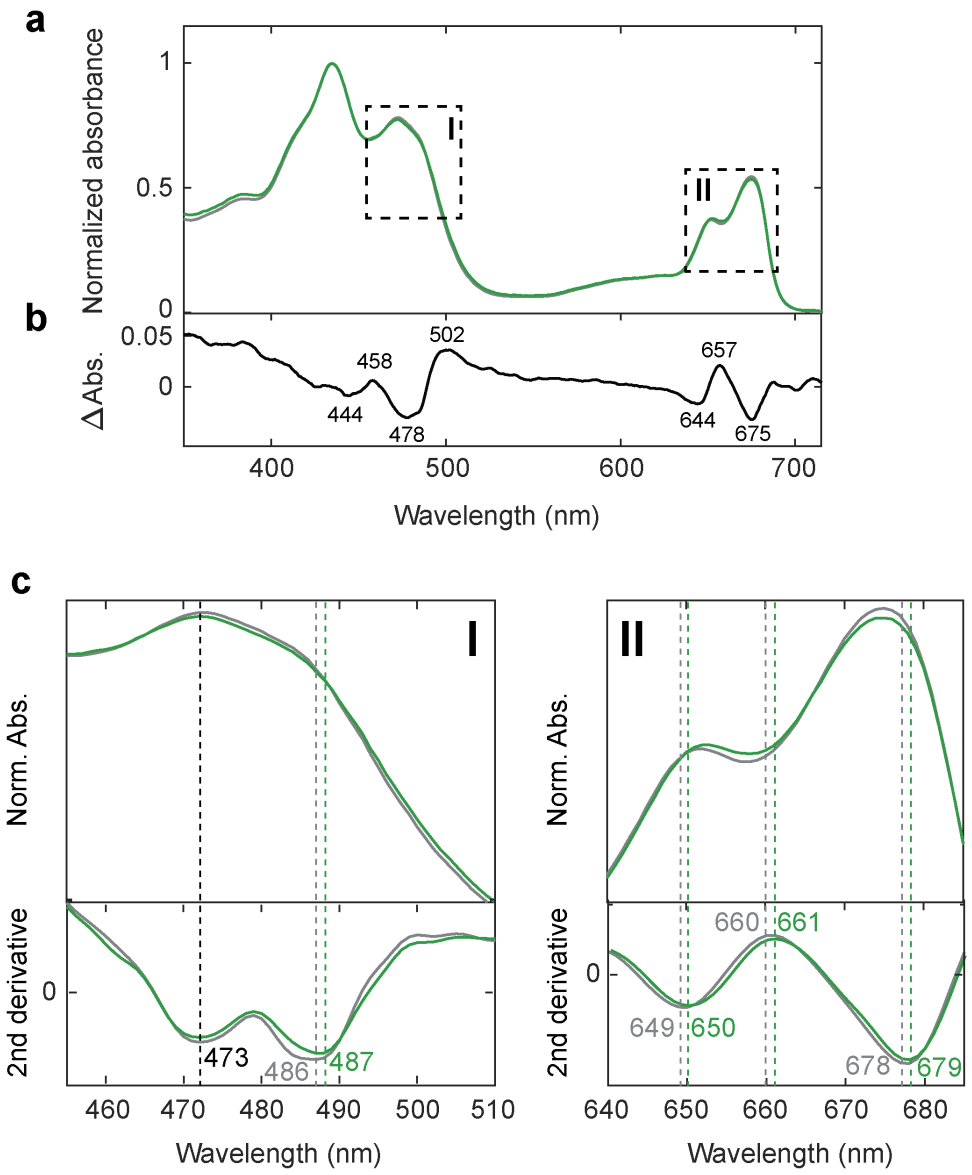

Supplementary Figure 11: Linear absorption of detergent-solubilized and disc-embedded LHCII. a, Linear absorption of LHCII in detergent (gray) and in soy asolectin nanodiscs (green), normalized to the 650 nm peak. **b**, Difference absorbance spectrum obtained by subtracting the normalized detergent spectrum from that of the nanodisc. **c**, Zoom-ins of the Car S_2 and Chl Q_y regions from **a** (labeled with dashed boxes). Bottom panels show the second derivative of the top panels with peak positions labeled.

Supplementary Note 5. Supplementary CD data

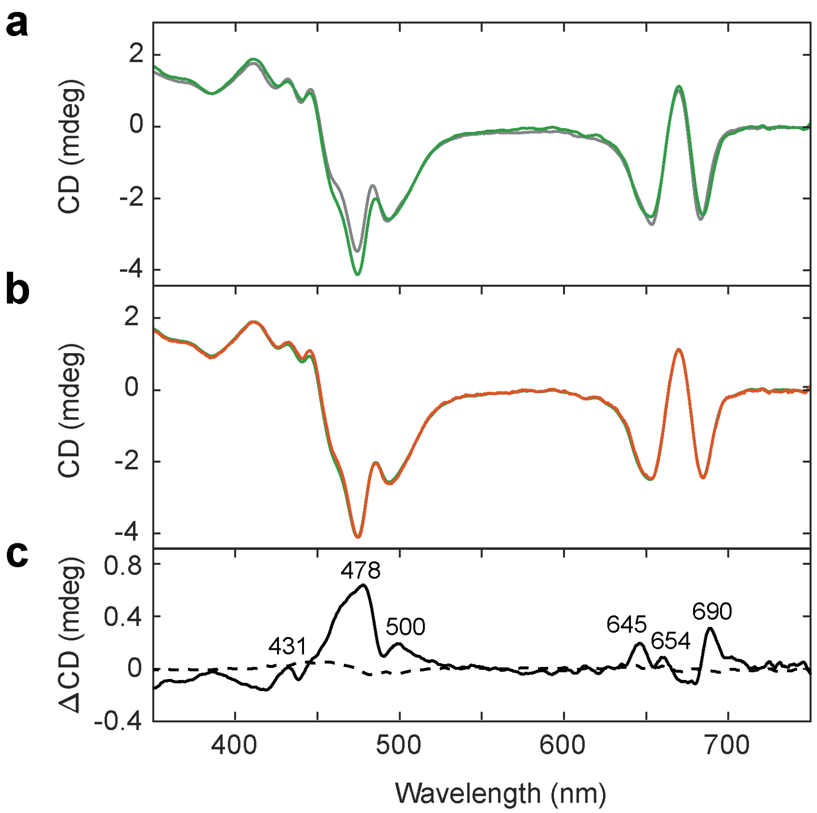

Supplementary Figure 12: Comparison of CD spectra in different environments. a, Comparison between detergent-solubilized (gray) and disc-embedded LHCII (green) with soy asolectin. The spectra are normalized to the sample absorbance at 675 nm. **b**, Comparison between soy asolectin discs (green) and native thylakoid lipid discs (orange, 50% MGDG, 27% DGDG, 12% SQDG and 11% PG). The spectra are normalized to the sample absorbance at 675 nm. **c**, Difference CD between detergent and soy asolectin discs in **a** (solid line) showing the effect of membrane environment. Dashed line shows the difference CD between soy asolectin discs and native thylakoid lipid discs (in **b**), indicating independence of the CD response on the lipid mixture used.

Supplementary Note 6. Fluorescence data

6.1 Steady-state fluorescence

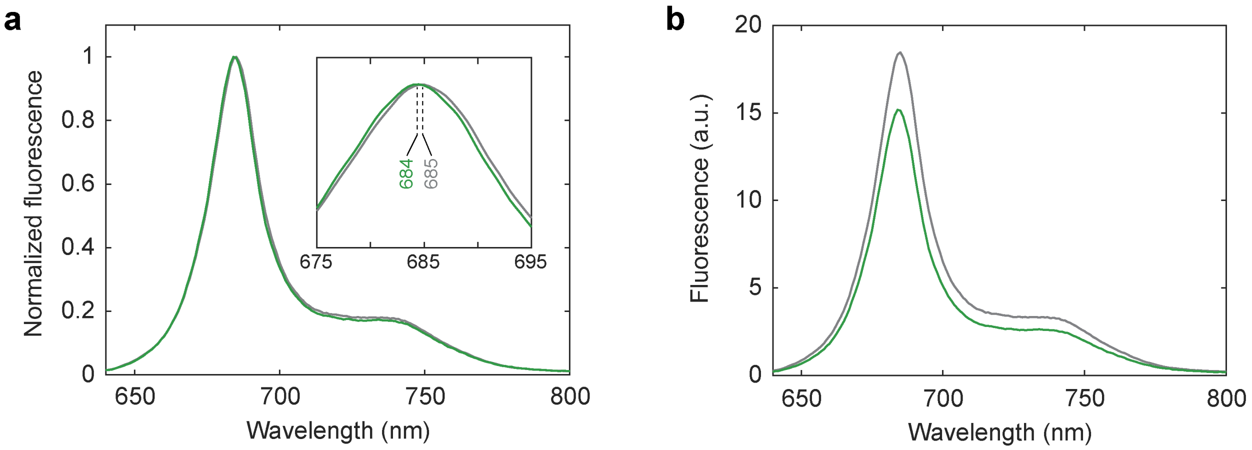

Supplementary Figure 13: Fluorescence spectra of detergent-solubilized and disc-embedded LHCII. Fluorescence spectra of LHCII in detergent (gray) and in soy asolectin nanodiscs (green), normalized to the maximum fluorescence peak (**a**) and to the OD at the excitation wavelength (630 nm) for comparison of fluorescence quantum yield (**b**). Inset of **a** displays zoom-in of the 675 – 695 nm range to show the shift in peak wavelength. In **b**, the integrated area of fluorescence in discs was 83% of that in detergent.

6.2 Time-resolved fluorescence

6.2.1 Comparison of fluorescence lifetimes between detergent-solubilized and disc-embedded LHCII

Excited-state dynamics on slower (hundreds of ps – ns) timescales were interrogated by TCSPC. While the fluorescence decay of LHCII in detergent was mono-exponential with a 3.4 ns time constant, LHCII incorporated into nanodiscs exhibited an additional, shorter (0.3 ps) component (Supplementary Figure 14a and Supplementary Table 3). The average fluorescence lifetime ($< \tau_{\mathrm{fl}} >$) was reduced in discs by 18%, from 3.4 to 2.8 ns. Our results are consistent with previously reported results on nanodiscs produced with the same membrane scaffolding protein and lipids.[6]

6.2.2 Effect of lipid environment on the fluorescence lifetimes in the membrane

To examine if the observed difference in the fluorescence dynamics is dependent on which lipids are used to form nanodiscs, we compared the fluorescence decay profiles of nanodiscs with soy asolectin lipid and native thylakoid lipid mixtures (see Supplementary Note 1.2 for details of the different lipids used). As shown in Supplementary Figure 14b, the fluorescence decay profiles are identical for nanodiscs regardless of the lipids used. Thus, we conclude that the difference in fluorescence lifetimes is caused by introduction of the membrane environment, not by specific protein-lipid interactions.

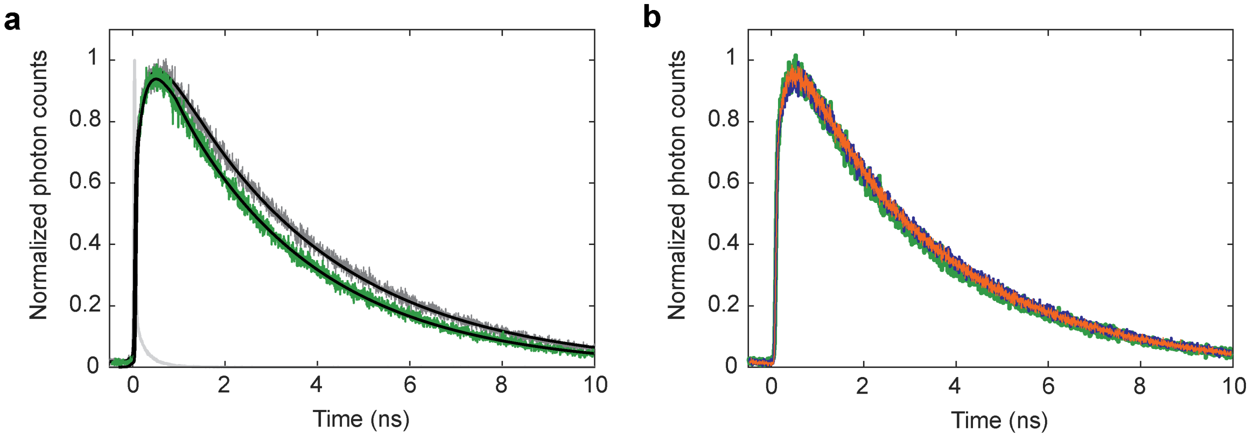

Supplementary Figure 14: Fluorescence lifetime comparison of detergent-solubilized and disc-embedded LHCII. a, Fitted fluorescence decay profiles of LHCII solubilized in detergent (gray) and embedded in nanodiscs with soy asolectin lipid (green). IRF is shown in light gray. The fitted parameters are shown in Supplementary Table 3. **b**, Overlay of the TCSPC profiles of LHCII nanodiscs with different lipid conditions. Green: soy asolectin, blue: 62% DGDG, 17% SQDG, 21% PG, orange: 50% MGDG, 27% DGDG, 12% SQDG and 11% PG.

Supplementary Table 3: Fitted parameters from fluorescence lifetime measurements.

Sample	A_1[a]	τ_1 (ns)[b]	A_2	τ_2 (ns)	$< \tau_{fl} >$[c]
Detergent-solubilized	–	–	1.00	3.4	3.4
Discs (soy asolectin)	0.09	0.3	0.91	3.1	2.8
Discs (50% MGDG, 27% DGDG, 12% SQDG and 11% PG)	0.10	0.3	0.90	3.1	2.8
Discs (62% DGDG, 17% SQDG, 21% PG)	0.10	0.3	0.90	3.1	2.8

[a] Normalized amplitude in percentage.

[b] Fitted time constant.

[c] Average fluorescence lifetime calculated by weighted average of the fitted components.

Supplementary Note 7. Supplementary 2DES data

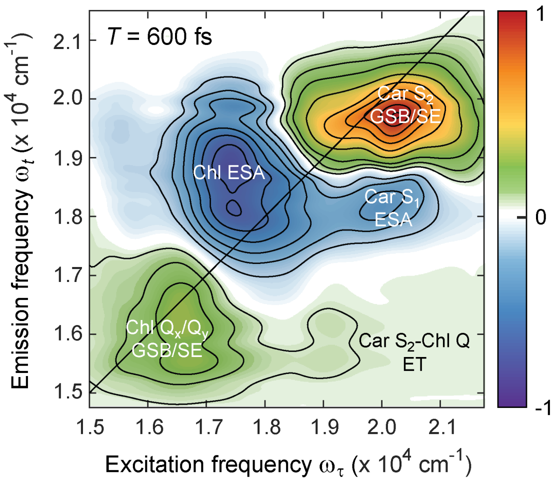

Supplementary Figure 15: Representative ultrabroadband 2D spectrum of LHCII. Absorptive 2D spectrum at T = 600 fs (in detergent, measured with laser spectrum 1) with the main spectral features labeled with text.

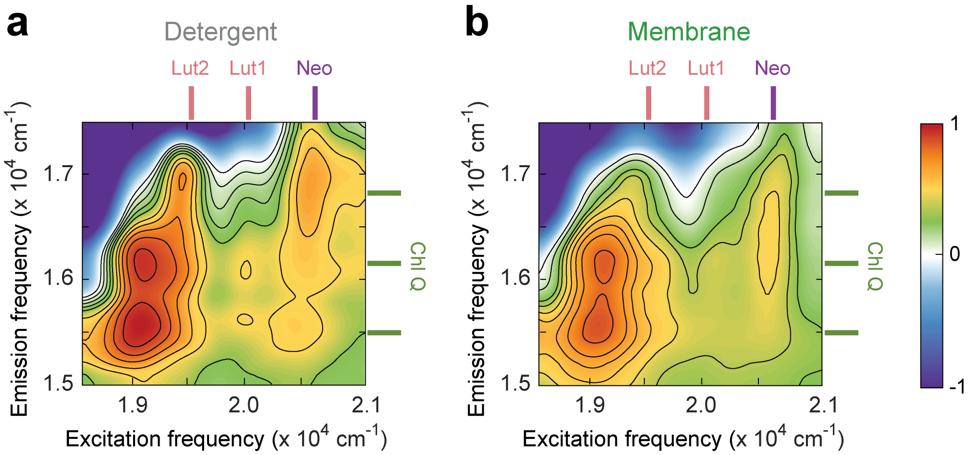

Supplementary Figure 16: Car S_2-Chl Q cross peaks. Absorptive 2D spectrum at T = 300 fs in the Car-Chl cross peak region (**a**, detergent; **b**, membrane).

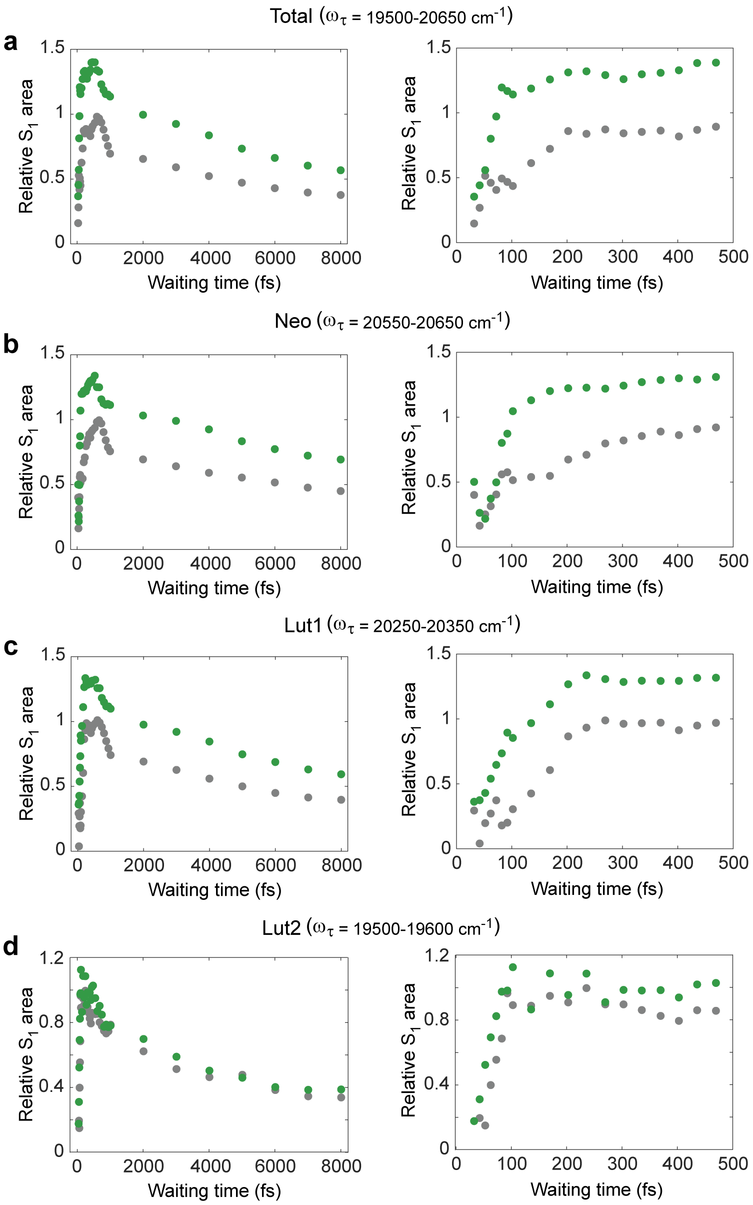

Supplementary Figure 17: Relative area of Car S_1 ESA normalized to the initial S_2 population. Traces in detergent and in membrane discs are shown in gray and green, respectively. Relative S_1 areas are calculated by normalizing the S_1 ESA intensity at each T to the initial S_2 GSB intensity at $T = 30$ fs. Right panels show zoom-ins of the initial 500 fs. **a** shows the ratio between the total integrated area using the broad ω_τ range as indicated in the figure. **b**-**d** are calculated with narrower ω_τ ranges to account for the response from the three individual Cars. Fixed ranges of $\omega_t = 19,200 - 21,000$ cm^{-1} (S_2 GSB, for both detergent and membrane) and $17,500 - 18,700$ cm^{-1} (S_1 ESA, detergent)/$17,700 - 18,900$ cm^{-1} (S_1 ESA, membrane) were used.

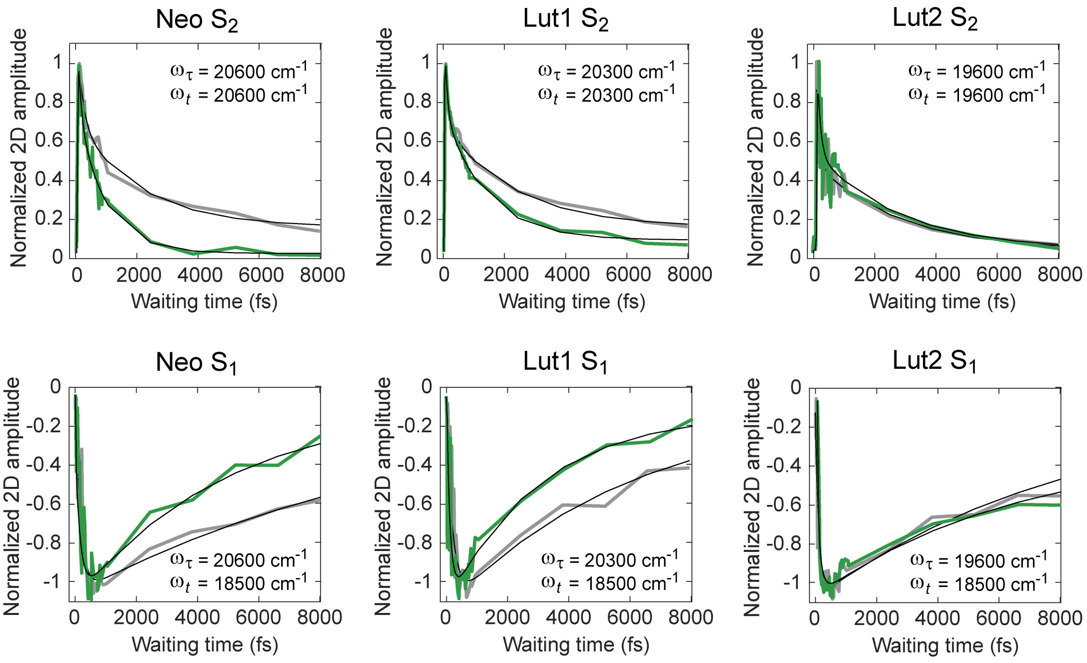

Supplementary Figure 18: Waiting time traces of Car S_2/S_1 peaks. Gray and green traces correspond to LHCII in detergent and in membrane discs, respectively. All traces were generated by integrating the 2D intensity over frequency intervals of 100 cm^{-1} (ω_τ) $\times$ 100 cm^{-1} (ω_t) around the center frequencies specified in each figure. Fit parameters are listed in Supplementary Table 4 below.

Supplementary Table 4: Fitted parameters from Car S_2/S_1 region of 2D spectra.

		A_1[a]	τ_1 (fs)[b]	A_2	τ_2 (fs)
Detergent	Neo S_2	0.42	115 ± 10	0.58	3,040 ± 300
	Lut1 S_2	0.32	110 ± 25	0.68	2,500 ± 270
	Lut2 S_2	0.56	80 ± 25	0.44	2,630 ± 570
	Neo S_1	-0.46	200 ± 50	0.54	10,000 ± 2,660
	Lut1 S_1	-0.36	160 ± 30	0.64	6,220 ± 1,080
	Lut2 S_1	-0.25	135 ± 27	0.75	7,100 ± 1,900
Disc	Neo S_2	0.49	63 ± 8	0.51	1,255 ± 150
	Lut1 S_2	0.38	100 ± 20	0.62	1,458 ± 138
	Lut2 S_2	0.50	88 ± 17	0.50	2,600 ± 550
	Neo S_1	-0.49	130 ± 33	0.51	4,620 ± 1,000
	Lut1 S_1	-0.42	110 ± 25	0.58	2,900 ± 600
	Lut2 S_1	-0.30	125 ± 25	0.70	7,150 ± 1,860

[a] Normalized amplitude in percentage. Negative amplitude indicates exponential rise.

[b] Fitted time constant with 90% confidence interval.

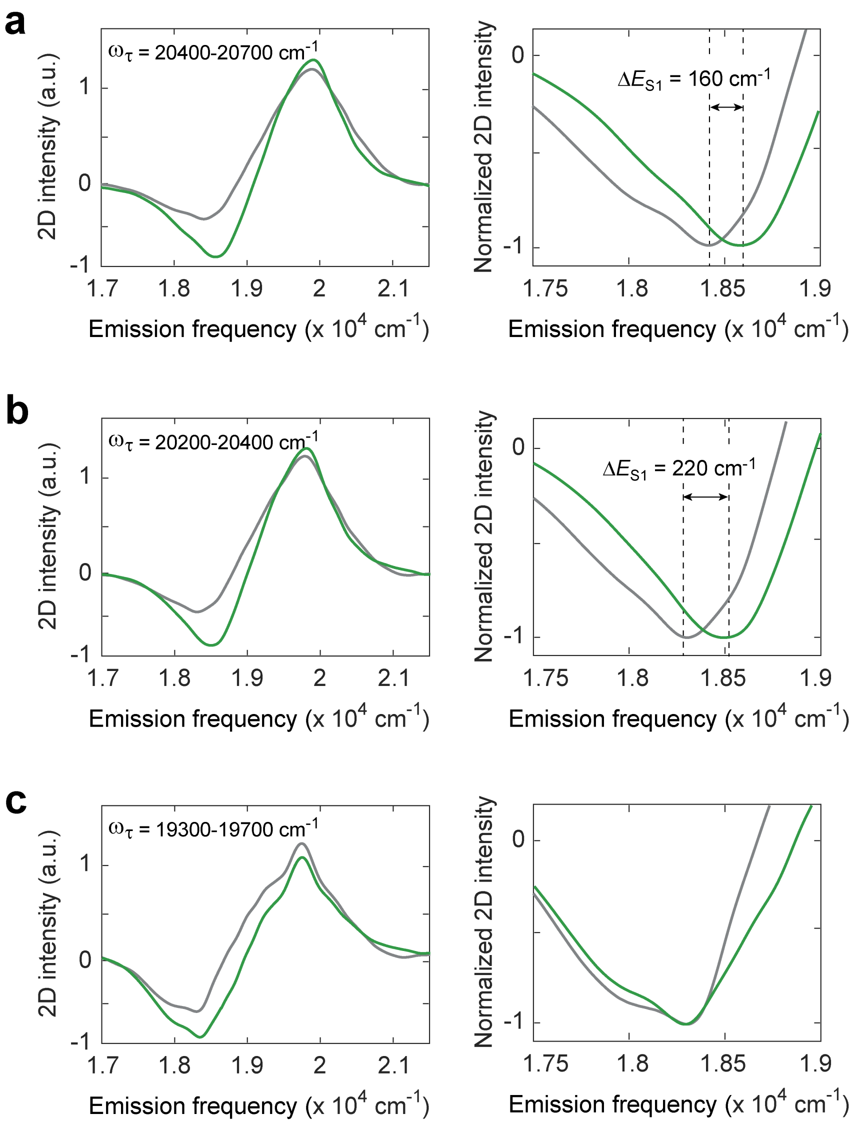

Supplementary Figure 19: S_1 energy shift of the Cars. Projection of the 2D spectra at $T = 500$ fs onto the ω_t axis for ω_τ ranges indicated inside each figure on the left column, which are the excitation frequencies for Neo (**a**), Lut1 (**b**), and Lut2 (**c**), respectively. Right column shows a closer view of the ESA, where both traces are normalized to the same scale. Gray and green traces correspond to LHCII in detergent and in membrane discs, respectively.

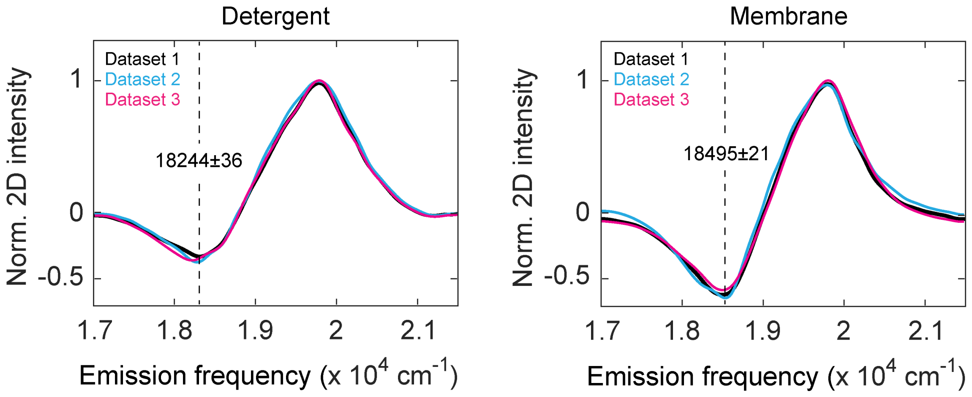

Supplementary Figure 20: Reproducibility of the S_1 energy shift of the Cars. Overlay of three replicates of the $T = 500$ fs projection traces in detergent (left) and in the membrane (right). Both panels are normalized to the positive maximum of the 2D intensity. The frequency value in each panel shows the average peak frequency and error retrieved from the replicates.

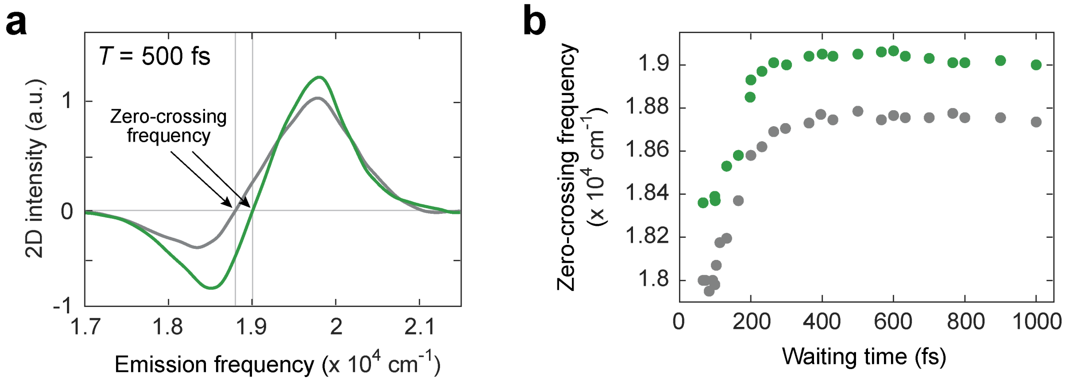

Supplementary Figure 21: Vibrational cooling dynamics of the Cars. a is a replicate of Fig. 2f in the main text with guidelines (light gray) added to show the definition of the zero-crossing frequency. **b** shows the evolution of the zero-crossing frequency over time for the first 1,000 fs. Gray trace corresponds to detergent-solubilized and green to disc-embedded sample, respectively.

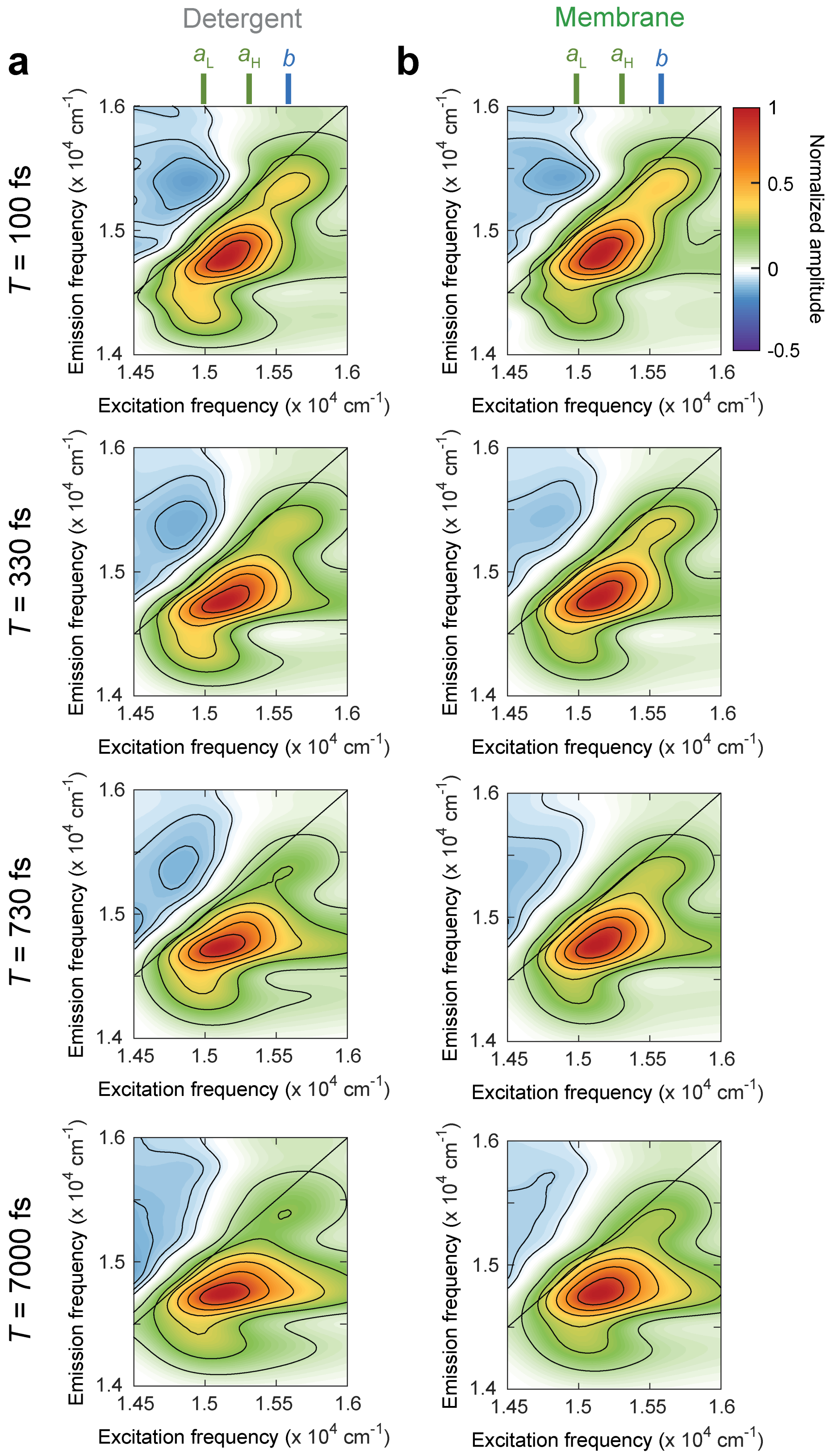

Supplementary Figure 22: Absorptive 2D spectra of Chl Q_y region at selected waiting times. a displays detergent-solubilized and **b** membrane-embedded LHCII. All spectra are normalized to the same scale. Contour lines are drawn at 15% and 5% intervals for positive and negative signal, respectively.

7.1 Peak assignments in the Chl Q_y region

In agreement with previously reported results,[20, 21] the 2D spectra in this region show diagonal peaks at the energies of the Q_y bands of Chl *b* and Chl *a* (peaks 1–3, Supplementary Figure 23). The pronounced elongation of the Chl *a* peak implies the presence of the higher-energy (a_H) and lower-energy pools (a_L) of Chl *a*, which are resolved into two separate peaks at cryogenic temperatures but not at room temperature (Supplementary Table 2).[20] At the intersections of the frequencies of 1–3, cross peaks (peaks 4–6) grow in at early waiting times as energy migrates from Chl *b* to the lower-lying Chl *a* pools. Our data also contain non-negligible contribution from the SE of Chl *a* at $\omega_t < 14{,}500\ \text{cm}^{-1}$ due to the broad bandwidth of our laser spectrum that covers part of the Chl fluorescence.[22]

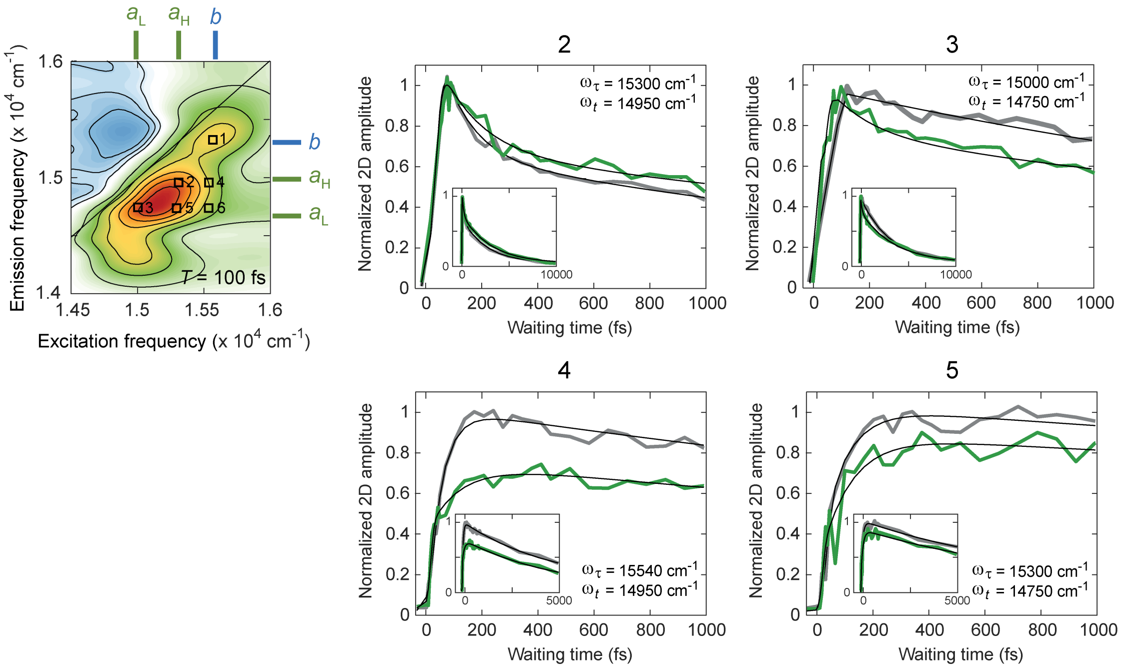

Supplementary Figure 23: Waiting time traces of Chl Q_y peaks. Gray and green traces correspond to LHCII in detergent and in membrane discs, respectively, and black lines are the fit curves. Peak positions are labeled in the $T = 100$ fs spectrum (detergent) shown on the left. All traces were generated by integrating the 2D intensity over frequency intervals of $100\ \text{cm}^{-1}$ (ω_τ) $\times$ $100\ \text{cm}^{-1}$ (ω_t) around the center frequencies specified in each figure. Insets show the longer-timescale dynamics for $T = 0 - 10{,}000$ fs (peaks 2, 3) and $0 - 5{,}000$ fs (peaks 4, 5). Fit parameters are listed in Supplementary Table 5 below. Waiting time traces of peaks 1 and 6 are shown in the main text, in Fig. 3b, c.

Supplementary Table 5: Fitted parameters from Chl Q_y region of 2D spectra.

		A_1[c]	τ_1 (fs)[d]	A_2	τ_2 (fs)
Detergent	1 (Chl b Q_y)	0.67	240 ± 20	0.33	2,950 ± 360
	2 (Chl a_H Q_y)	0.33	168 ± 20	0.67	2,670 ± 350
	3 (Chl a_L Q_y)	–	–	1.00	3,085 ± 400
	4 ($b \rightarrow a_H$ transfer)	-0.48	80 ± 20	0.52	4,400 ± 600
	5 ($a_H \rightarrow a_L$ transfer)	-0.38	90 ± 23	0.62	9,550 ± 2,500
	6 ($b \rightarrow a_L$ transfer)	-0.47	130 ± 20	0.53	8,300 ± 1,600
	Chl SE [a]	0.39	350 ± 30	0.61	4,100 ± 450
	Car S_1 ESA[b]	-0.33	300 ± 60	0.67	7,645 ± 500
Disc	1 (Chl b Q_y)	0.58	400 ± 35	0.42	2,970 ± 340
	2 (Chl a_H Q_y)	0.33	165 ± 20	0.67	2,930 ± 420
	3 (Chl a_L Q_y)	0.39	195 ± 30	0.61	2,600 ± 375
	4 ($b \rightarrow a_H$ transfer)	-0.37	132 ± 22	0.63	4,900 ± 950
	5 ($a_H \rightarrow a_L$ transfer)	-0.40	105 ± 20	0.60	10,400 ± 2,500
	6 ($b \rightarrow a_L$ transfer)	-0.38	225 ± 20	0.62	8,680 ± 1,300
	Chl SE [a]	0.53	270 ± 20	0.47	3,270 ± 300
	Car S_1 ESA[b]	-0.45	220 ± 50	0.55	3,810 ± 350

[a] Integrated over the area ω_τ = 14,775 – 15,075 cm^{-1}, ω_t = 14,270 – 14,670 cm^{-1} (see Fig. 3d in the main text for the waiting time traces).

[b] Integrated over the area ω_τ = 14,775 – 15,075 cm^{-1}, ω_t = 18,200 – 18,600 cm^{-1} (see Fig. 3e in the main text for the waiting time traces).

[c] Normalized amplitude in percentage. Negative amplitude indicates exponential rise.

[d] Fitted time constant with 90% confidence interval.

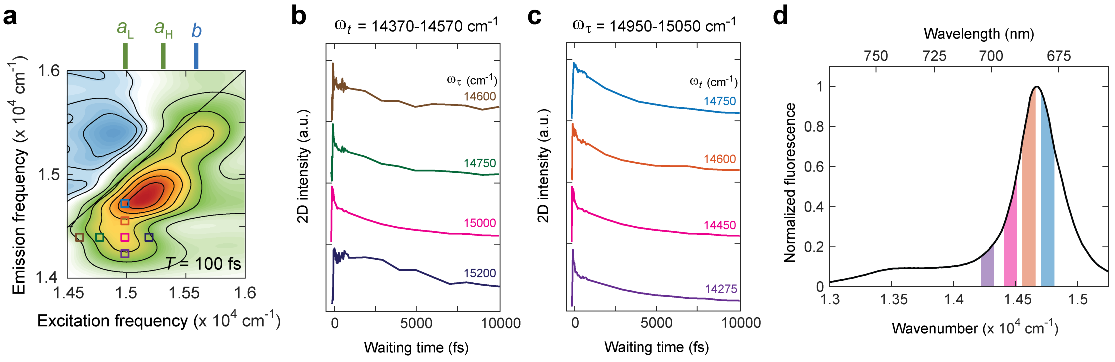

Supplementary Figure 24: Frequency dependence of the low-energy Chl *a* decay. Waiting time traces of terminal Chl *a* SE monitored as a function of excitation frequency (**b**, ω_t fixed at $14,370 - 14,570$ cm^{-1}) and emission frequency (**c**, ω_τ fixed at $14,950 - 15,050$ cm^{-1}). The peak positions at which the time traces are plotted are labeled with color-coded open squares in **a**, and indicated in **b** and **c**. All traces are normalized to the same scale, and vertically offset for clarity. Only detergent data are shown, but the same trends were observed in nanodiscs. **d**, Overlay of the steady-state fluorescence spectrum of LHCII with the probe ranges shown in **c**.

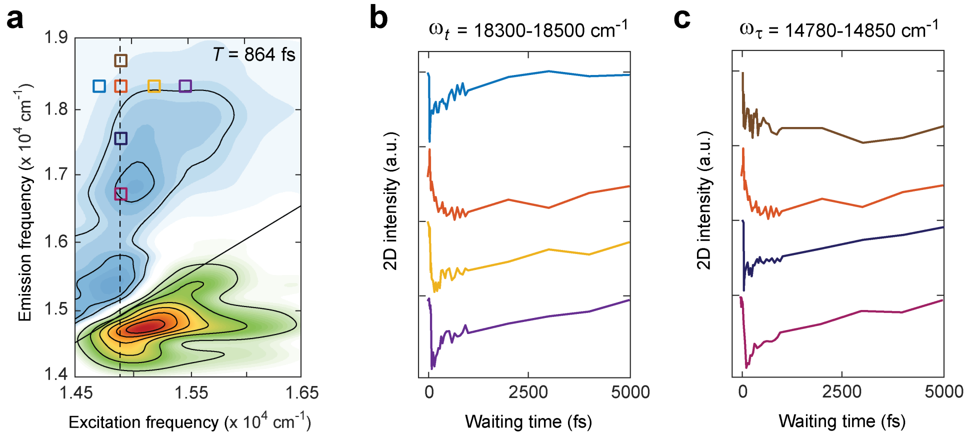

Supplementary Figure 25: Waiting time traces of Chl *a* ESA. Waiting time traces of ESA monitored as a function of excitation frequency (**b**, ω_t fixed at $18,300 - 18,500$ cm^{-1}) and emission frequency (**c**, ω_τ fixed at $14,780 - 14,850$ cm^{-1}). The peak positions at which the time traces are plotted are labeled with color-coded open squares in **a**. All traces are normalized to the same scale, and vertically offset for clarity. Only detergent data are shown, but the same trends were observed in nanodiscs.

7.2 Fitting and assignment of the picosecond component (τ_2)

The waiting time (T) was scanned from 1–10 ps, which is sufficient to characterize processes up to a few picoseconds. On the longer timescale in LHCII, there are picosecond vibrational relaxation processes occurring as well as the nanosecond timescale of the Chl fluorescence.[23–26] Therefore, we collectively fit the slower processes as a single long-timescale component, τ_2 (Supplementary Table 5).

We show below that the long component can be fit reasonably well with vastly different time constants due to the limited temporal range. As shown in Supplementary Figure 26, changing τ_2 from 4,100 fs, the best fit parameter, to 20,000 fs only slightly affects the goodness of the fit (R^2). Furthermore, the amplitude A_2 changes along with the change in τ_2, from 61% to 80%. These results highlight that neither the time constant nor the amplitude of the long-timescale dynamics is well characterized within our temporal range.

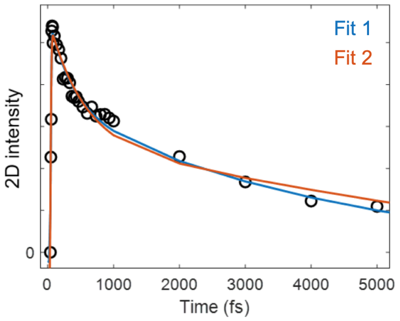

Fit parameters and goodness of the fit

	A_1 (%)	τ_1 (fs)	A_2 (%)	τ_2 (fs)	R^2
Fit 1	39	350	61	4100	0.963
Fit 2	20	322	80	20000	0.947

Supplementary Figure 26: Comparison of fits with different τ_2 values. Left panel shows the comparison of two different fits of the low-energy Chl *a* decay in detergent (gray curve in Fig. 3d in the main text). Fit 1 (blue): Best fit (as shown in Fig. 3d and Supplementary Table 5), fit 2 (orange): an alternative fit where τ_2 is fixed to 20,000 fs. The fit parameters and goodness of the fit are summarized in the table on the right.

Supplementary Note 8. Kinetic models

A set of coarse-grained kinetic models was constructed to simulate the dynamics observed in the experimental data. Detailed descriptions of each model and comparison with experimental data are provided below.

8.1 Branching ratio of the Car S_2 population

The kinetic scheme for the Car S_2 state is shown in Supplementary Figure 27a. The excited-state population on the S_2 state of the Cars decays via two channels: internal conversion to the S_1 state (k_{21}) and energy transfer to Chl Q states (k_{2q}). $k_{d'}$ and k_d denote deexcitation rate constant of the Car S_1 and Chl Q population, respectively. The differential equations that describe the temporal evolution of populations are

$$\dot{P_{S_2}} = -(k_{21} + k_{sq})P_{S_2}$$
$$\dot{P_{S_1}} = k_{21}P_{S_2} - k_{d'}P_{S_1}$$
$$\dot{P_Q} = k_{2q}P_{S_2} - k_dP_Q$$

The initial populations are estimated based on the extinction coefficient and laser intensity (spectrum 1 in Supplementary Figure 7) at the absorption wavelength range of the Cars and Chls, and scaled such that $P_{S_2} + P_{S_1} + P_Q = 1$ at $T = 0$. Pathways involving the terminal Chl *a* locus were excluded given the negligible probability for direct Chl *a* excitation with spectrum 1.

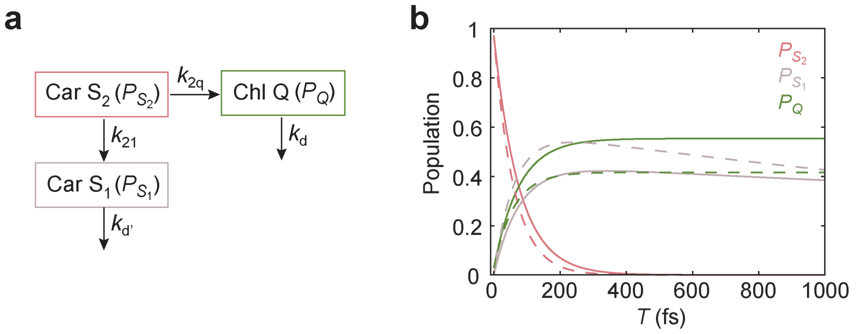

Supplementary Figure 27: Kinetic model for Car S_2 branching pathways. a, Kinetic scheme showing the relevant pigment states and rate constants. **b**, Time evolution of the population on each state, calculated using the experimental rate constants obtained for detergent-solubilized (solid) and membrane-embedded LHCII (dashed curves). k_{21} = (160 fs)$^{-1}$/(110 fs)$^{-1}$, k_{2q} = (150 fs)$^{-1}$/(150 fs)$^{-1}$, k_d = (3.4 ns)$^{-1}$/(2.8 ns)$^{-1}$, $k_{d'}$ = (6.22 ps)$^{-1}$/(2.9 ps)$^{-1}$ (detergent/membrane).

Supplementary Figure 27b shows the temporal evolution of populations using the rate constants obtained for detergent-solubilized LHCII (solid lines) and those obtained for membrane-embedded LHCII (dashed lines). Consistent with the 35(±9)% decrease in the Car-Chl cross peak intensity (Fig. 2d, e in the main

text), Chl Q population decreases by 28%. Car S_1 population increases by 30%, also consistent with the increase in Car S_1 ESA intensity in the membrane. Varying the rate constants did not result in any significant changes to the population dynamics. The dynamics are insensitive to changes in the two slow rate constants k_d and $k_{d'}$. Varying k_{21} from 150−200 fs (detergent)/110−130 fs (membrane) or k_{2q} from 100−150 fs resulted in $<$ 3% difference in the population, demonstrating the robustness of the model.

8.2 Branching of the Chl Q states and Chl-to-Car energy transfer

The kinetic scheme of the Chl Q states is shown in Supplementary Figure 28a, and the differential equations describing the temporal evolution of the populations are

$$\dot{P_b} = -(k_{ba} + k_{ba'})P_b$$

$$\dot{P_{a_H}} = k_{ba}P_b - k_{aa}P_{a_H}$$

$$\dot{P_{a_L}} = k_{aa}P_{a_H} + k_{ba'}P_b - k_dP_{a_L} - k_{a1}P_{a_L}$$

$$\dot{P_{S_1}} = k_{a1}P_{a_L} - k_{d'}P_{S_1}$$

The initial populations are estimated based on the extinction coefficient and laser intensity (spectrum 2 in Supplementary Figure 7) at the absorption wavelength range of the Cars and Chls, and scaled such that $P_b + P_{a_H} + P_{a_L} = 1$ at $T = 0$. Pathways involving the Car S_2 state were excluded given the negligible probability for direct Car S_2 excitation with spectrum 2.

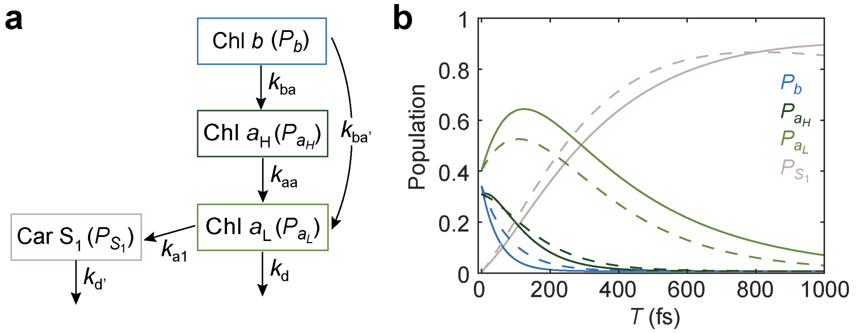

Supplementary Figure 28: Kinetic model for Chl branching and Chl-to-Car energy transfer. a, Kinetic scheme showing the relevant pigment states and rate constants. **b**, Time evolution of the population on each state, calculated using the experimental rate constants obtained for detergent-solubilized (solid) and membrane-embedded LHCII (dashed curves). k_{ba} = (80 fs)$^{-1}$/(130 fs)$^{-1}$, $k_{ba'}$ = (130 fs)$^{-1}$/(225 fs)$^{-1}$, k_{aa} = (90 fs)$^{-1}$/(105 fs)$^{-1}$, k_{a1} = (350 fs)$^{-1}$/(270 fs)$^{-1}$, k_d = (3.4 ns)$^{-1}$/(2.8 ns)$^{-1}$, $k_{d'}$ = (7.7 ps)$^{-1}$/(3.8 ps)$^{-1}$ (detergent/membrane).

Supplementary Figure 28b shows the temporal evolution of populations using the rate constants obtained from the 2D experiment. Varying k_{a1} from 300−350 fs (detergent)/220−270 fs (membrane) resulted in

$< 3\%$ difference in the population, demonstrating the robustness of the model. The relative populations on the terminal Chl *a* locus as well as the Car S_1 state in the membrane, are consistent between our kinetic model and experimental data with small deviations (Fig. 4b in the main text).

Supplementary References

1. Bassi, R. & Simpson, D. Chlorophyll-protein complexes of barley photosystem I. *FEBS J.* **163**, 221–230 (1987).
2. Gilmore, A. M. & Yamamoto, H. Y. Zeaxanthin formation and energy-dependent fluorescence quenching in pea chloroplasts under artificially mediated linear and cyclic electron transport. *Plant Physiol.* **96**, 635–643 (1991).
3. Bayburt, T. H., Grinkova, Y. V. & Sligar, S. G. Self-assembly of discoidal phospholipid bilayer nanoparticles with membrane scaffold proteins. *Nano Lett.* **2**, 853–856 (2002).
4. Ritchie, T. K. *et al.* Reconstitution of membrane proteins in phospholipid bilayer nanodiscs. *Methods Enzymol.* **464**, 211–231 (2009).
5. Morrow, J. A., Arnold, K. S. & Weisgraber, K. H. Functional characterization of apolipoprotein e isoforms overexpressed in *Escherichia coli*. *Protein Expr. Purif.* **16**, 224–230 (1999).
6. Pandit, A. *et al.* Assembly of the major light-harvesting complex II in lipid nanodiscs. *Biophys. J.* **101**, 2507–2515 (2011).
7. Crisafi, E. & Pandit, A. Disentangling protein and lipid interactions that control a molecular switch in photosynthetic light harvesting. *BBA-Biomembranes* **1859**, 40–47 (2017).
8. Boldog, T., Li, M. & Hazelbauer, G. L. Using nanodiscs to create water-soluble transmembrane chemoreceptors inserted in lipid bilayers. *Methods Enzymol.* **423**, 317–335 (2007).
9. Boekema, E. J. Negative staining of integral membrane proteins. *Micron Microscop. Acta* **22**, 361–369 (1991).
10. Douce, R. & Joyard, J. Biosynthesis of thylakoid membrane lipids. In Ort, D. R. & Yocum, C. F. (eds.) *Advances in Photosynthesis/Oxygenic Photosynthesis: The Light Reactions* (Kluwer Academic Publishers, 1996).
11. Moon, S. *et al.* Endotoxin-free purification of recombinant membrane scaffold protein expressed in *Escherichia coli*. *Process Biochem.* **66**, 230–236 (2018).
12. Schwille, P., Haupts, U., Maiti, S. & Webb, W. W. Molecular dynamics in living cells observed by fluorescence correlation spectroscopy with one-and two-photon excitation. *Biophys. J.* **77**, 2251–2265 (1999).
13. Trebino, R. *et al.* Measuring ultrashort laser pulses in the time-frequency domain using frequency-resolved optical gating. *Rev. Sci. Instrum.* **68**, 3277–3295 (1997).
14. Jonas, D. M. Two-dimensional femtosecond spectroscopy. *Annu. Rev. Phys. Chem.* **54**, 425–463 (2003).
15. Son, M., Mosquera-Vázquez, S. & Schlau-Cohen, G. S. Ultrabroadband 2D electronic spectroscopy with high-speed, shot-to-shot detection. *Opt. Express* **25**, 18950–18962 (2017).
16. Bello, O. D., Auclair, S. M., Rothman, J. E. & Krishnakumar, S. S. Using ApoE nanolipoprotein particles to analyze SNARE-induced fusion pores. *Langmuir* **32**, 3015–3023 (2016).
17. Ruban, A. V. *et al.* Identification of a mechanism of photoprotective energy dissipation in higher plants. *Nature* **450**, 575 (2007).
18. Son, M., Pinnola, A., Bassi, R. & Schlau-Cohen, G. S. The electronic structure of lutein 2 is optimized for light harvesting in plants. *Chem* **5**, 575–584 (2019).
19. Caffarri, S., Croce, R., Breton, J. & Bassi, R. The major antenna complex of photosystem II has a xanthophyll binding site not involved in light harvesting. *J. Biol. Chem.* **276**, 35924–35933 (2001).
20. Schlau-Cohen, G. S. *et al.* Pathways of energy flow in LHCII from two-dimensional electronic spectroscopy. *J.*

Phys. Chem. B **113**, 15352–15363 (2009).

21. Duan, H.-G. *et al.* Two-dimensional electronic spectroscopy of light-harvesting complex II at ambient temperature: A joint experimental and theoretical study. *J. Phys. Chem. B* **119**, 12017–12027 (2015).
22. Moca, R., Meech, S. R. & Heisler, I. A. Two-dimensional electronic spectroscopy of chlorophyll a: Solvent dependent spectral evolution. *J. Phys. Chem. B* **119**, 8623–8630 (2015).
23. Dong, L.-Q., Niu, K. & Cong, S.-L. Theoretical study of vibrational relaxation and internal conversion dynamics of chlorophyll-a in ethyl acetate solvent in femtosecond laser fields. *Chem. Phys. Lett.* **432**, 286–290 (2006).
24. Saccon, F. *et al.* Spectroscopic properties of violaxanthin and lutein triplet states in LHCII are independent of carotenoid composition. *J. Phys. Chem. B* **123**, 9312–9320 (2019).
25. Hughes, J. L., Conlon, B., Wydrzynski, T. & Krausz, E. The assignment of $Q_y(1, 0)$ vibrational structure and Q_x for chlorophyll *a*. *Phys. Procedia* **3**, 1591–1599 (2010).
26. Du, J., Teramoto, T., Nakata, K., Tokunaga, E. & Kobayashi, T. Real-time vibrational dynamics in chlorophyll *a* studied with a few-cycle pulse laser. *Biophys. J.* **101**, 995–1003 (2011).
